# Activation of POMC neurons to adiponectin participating in EA-mediated improvement of high-fat diet IR mice

**DOI:** 10.3389/fnins.2023.1145079

**Published:** 2023-03-21

**Authors:** Wanling Xu, Junfeng Li, Chang Ji, Danwei Fang, Lulu Yao, Nenggui Xu, Wei Yi

**Affiliations:** ^1^South China Research Center for Acupuncture and Moxibustion, Medical College of Acu-Moxi and Rehabilitation, Guangzhou University of Chinese Medicine, Guangzhou, China; ^2^The First Affiliated Hospital, Jinan University, Guangzhou, China

**Keywords:** electroacupuncture, ZuSanLi, insulin resistance, adiponectin, POMC

## Abstract

**Background:**

Insulin resistance (IR) is one of the common pathological manifestations of metabolic-related diseases, and the prevalence of relevant diseases is high. Acupuncture is beneficial to IR patients, but the central mechanism underlying this treatment remains unclear. This study provides mechanistic insights into how electroacupuncture (EA) improves IR through the response of Pro-opiomelanocortin (POMC) neurons to adiponectin (Adipo).

**Methods:**

Glucose tolerance tests (GTT), Insulin tolerance tests (ITT) and fasting blood glucose (FBG) were detected by glucometer. Serum insulin, Adipo and skeletal muscle adiponectin receptor 1 (AdipoR1) protein levels were examined by ELISA. Homeostasis model assessment estimated insulin resistance (HOMA-IR) was calculated using the following formula: HOMA-IR = fasting insulin (FINS) (mU/L) × FBG (mmol/L)/22.5. The expression levels of AdipoR1 and Adipo mRNA in skeletal muscle were detected by real-time PCR quantification. The co-marking of c-Fos/AdipoR1 and POMC neurons were investigated using immunofluorescence. Spontaneous excitatory postsynaptic currents (sEPSCs) of POMC neurons and the response of POMC neurons to Adipo were detected via electrophysiology.

**Results:**

EA significantly ameliorated HFD-induced impairment of GTT, ITT, FBG, and HOMA-IR which was correlated with recovery of the expression level of AdipoR1 and Adipo in skeletal muscle. The improved response of POMC neurons to Adipo in the hypothalamus may be a key factor in correcting abnormal glucose tolerance and improving IR.

**Conclusion:**

This study demonstrates that EA can ameliorate HFD-induced impaired glucose tolerance through improved response of POMC neurons to Adipo in the hypothalamus, providing insight into the central mechanism of improving IR through EA.

## Introduction

Insulin resistance (IR) is defined as the reduced sensitivity of target cells expressing insulin receptors to insulin (Petersen and Shulman, 2018). Diseases closely related to insulin resistance include diabetes, obesity, non-alcoholic fatty liver disease, polycystic ovary syndrome, and cardiovascular diseases, especially type 2 diabetes (Rice et al., 2005; Mojiminiyi et al., 2007; Unlühizarci et al., 2007; Diamanti-Kandarakis and Dunaif, 2012; Nakata et al., 2016; Wu et al., 2016; Dahan and Reaven, 2019; Lee et al., 2020; Stener-Victorin et al., 2020). Globally, the prevalence of diabetes is expected to increase from 382 million in 2013 to 592 million in 2035, with type 2 diabetes accounting for approximately 90-95% of all cases (Laakso and Kuusisto, 2014). Metformin induces energy stress by inhibition of complex I of the mitochondrial respiratory chain, leading to changes in the ratio of ATP to AMP and activation of classically related protein kinases, which are key molecules in the regulation of metabolism and thus impact IR (Scherer et al., 1995). Direct activation of relevant protein kinases can recover IR in a variety of animal models (Fullerton et al., 2013; Cokorinos et al., 2017). However, inactivation of related protein kinases in the liver has no effect on the efficacy of drugs, while muscle-specific loss of related protein kinases rescues the effect of drugs. Therefore, muscle-associated protein kinases have been established as the main therapeutic target of IR (Cokorinos et al., 2017), suggesting that muscles may play an important role in improving insulin resistance.

Adipo is a hormone mainly secreted by adipocytes. By acting on key organs, including muscle tissue, pancreas, liver, adipose tissue, and brain, Adipo regulates glucose metabolism and insulin action. It can increase insulin sensitivity and reduce plasma concentration associated with insulin resistance (Fang and Judd, 2018). In IR, the level of Adipo in the blood is generally impaired (Hung et al., 2008; Sull et al., 2009). Regarded as insulin sensitizers, thiazolidinedione drugs (TZDs) can increase the ability of peripheral tissues to clear glucose, thus improving glucose metabolism (Miles et al., 2003; Zinman et al., 2009). The decrease of Adipo in rodents is related to decreased insulin response and high glucose levels, while systemic Adipo treatment or transgenic Adipo overexpression can reduce blood glucose (Qi et al., 2004). Additionally, TZD called pioglitazone can significantly improve IR in diabetic *ob/ob* mice, but not in Adipo-deficient *ob/ob* mice (Kubota et al., 2006). Adipo expression and number of mitochondria were decreased in the adipose tissue of obese mice, and these changes were reversed by rosiglitazone (Koh et al., 2007). Taken together, these indicate that Adipo has a therapeutic effect on insulin resistance.

Alongside its aforementioned role, Adipo also stimulates the activity of hypothalamic-related protein kinases (Yamauchi and Kadowaki, 2008). POMC neurons are key regulatory factors of metabolism and reproduction in the arcuate nucleus (ARC) of the hypothalamus and play a fundamental role in food and metabolism regulation. AdipoR1 is present in POMC neurons (Guillod-Maximin et al., 2009). A low molecular form of Adipo can cross the brain-blood barrier (BBB) and is also found in human cerebrospinal fluid (Kubota et al., 2007). Adipo enters the cerebrospinal fluid from the circulation, increases the activity of related protein kinases in the hypothalamus, and increases food intake through AdipoR1 (Thundyil et al., 2012). Under starvation conditions, Adipo triggers related protein kinases in the hypothalamus to promote food uptake (Yamauchi and Kadowaki, 2008). After intracerebroventricular injection of Adipo in mice, energy consumption is increased (Guillod-Maximin et al., 2009), food intake (Coope et al., 2008), and body weight are decreased. Adipo level correlates with the excitatory or inhibitory effect of Adipo on POMC neurons activity and feeding in ARC (Suyama et al., 2016). Thus, the peripheral and central roles of Adipo are closely related to energy metabolism and food intake. However, current studies have focused on peripheral target organs and the level of blood factors, while the central system mainly focuses on the influence of Adipo on food intake through POMC neurons and the participation of other pathway factors. Whether Adipo signal impacts the activity of peripheral skeletal muscle protein kinase and glucose absorption through the regulation of POMC neurons, and whether the regulation of Adipo signal by POMC neurons plays a key role in improving insulin resistance remain unclear.

Experimental studies have shown that acupuncture can correct various metabolic disorders such as hyperglycemia, overweight, hyperlipidemia, inflammation, altered sympathetic nervous system activity, and defective insulin signaling (Benrick et al., 2014; Lin et al., 2014; Li et al., 2018; Huang et al., 2020), all of which contribute to the improvement of IR. Lin (Lin et al., 2014) hypothesized that ST36 EA treatment stimulates the secretion of insulin-sensitive substances by releasing acetylcholine, and improves glucose tolerance and insulin activity by impacting insulin-sensitive target organs (chiefly muscles). Our previous study (Huang et al., 2016) demonstrated that acupuncture is capable of improving fasting blood glucose (FBG), plasma fasting insulin (FINS), insulin resistance index (HOMA-IR), and insulin signal molecules in OLETF rats. In this study, molecular biology, electrophysiology, and other experimental methods were used to observe the changes of peripheral molecular biology and the effects of central Adipo on POMC neurons activity in IR mice by EA, and to explore the mechanism of Adipo participating in EA to improve IR.

## Materials and methods

### Animals

Male C57BL/6J mice (10–14g; aged 4 weeks) were supplied by the Beijing Hua Fukang Biotechnology Co., LTD., and managed by the South China Research Center for Acupuncture and Moxibustion (Guangzhou, China). Mice were kept in a temperature-controlled (22–24°C, 50-70% humidity range) holding room with 12h light/dark cycle. Mice were given free access to water and food. The protocols were approved by the Ethics Committee of Guangzhou University of Chinese Medicine (No. 20210604003).

### Establishment of IR model in mice

After 3 days of adaptive feeding, mice were randomly divided into 2 groups, CD and HFD groups. Mice in CD and HFD groups were fed a control standard diet [20% kcal protein, 70% kcal carbohydrate and 10% kcal fat, H10010, Beijing HFK BIOSCIENCE CO.,LTD.) or high-fat diet (20% kcal protein, 20% kcal carbohydrate and 60% kcal fat, H10060, Beijing HFK BIOSCIENCE CO.,LTD.)], respectively. Body weights were measured once a week. After 8 weeks of feeding, mice were randomly selected from the CD group and HDF group for examination via glucose tolerance test (GTT), insulin tolerance test (ITT), fasting blood glucose (FBG), HOMA-IR, and serum insulin levels to establish the model.

### Electroacupuncture (EA) treatment

After the establishment of an IR model, the HFD group was randomly divided into an HFD group and an HFD + EA group. The mice were anesthetized by isoflurane and kept under anesthesia during EA treatment. The HFD group only received anesthesia without electroacupuncture. Disposable sterile acupuncture needles (0.16 mm × 7 mm; Beijing Zhongyan Taihe Medical InstrumentCp. Ltd., Tianjin, China) were parallelly inserted at a depth of 4-5 mm into the murine equivalent of the human ZuSanLi (ST36, about 3mm below the knee joint in the tibialis anterior muscle) acupoints bilaterally. The needle handles were connected to Huatuo electroacupuncture instrument. The EA stimulation was applied for 20 min with a dense-dispersed wave of 2Hz. Two groups were treated 6 days every week for 4 weeks.

### Glucose tolerance tests (GTT)

Glucose tolerance tests were performed on 12h-fasted animals. After determination of fasting blood glucose (FBG) levels at the end of mouse tails at 9 a.m., each animal received an intraperitoneal injection of 50% glucose (10 mL/kg) (G7201, Sigma). Blood glucose levels were measured after 30, 60, 90, and 120 min.

### Insulin tolerance tests (ITT)

Glucose tolerance tests were performed on 12h-fasted animals. After determination of fasting blood glucose (FBG) levels at the end of mouse tails at 9 a.m., each animal received an intraperitoneal injection of 1U/mL Insulin (10 mL/kg) (25R, Lilly France). Blood glucose levels were measured after 30, 60, 90, and 120 min.

### Serum analyses

All mice fasted for 12 h after prior acupuncture. On the second morning, fasting blood glucose (FBG) levels were tested from mouse tails. Mice were then anesthetized with tribromoethanol and blood samples were collected by eye enucleation. Samples were measured by ELISA to detect fasting insulin (FINS) (E-EL-M1382c; Elabscience, Wuhan, China) and Adipo (E-EL-M0002c; Elabscience, Wuhan, China). ELISA was performed according to the manufacturer’s instructions. Additionally, the following formula was used to calculate the homeostasis model assessment estimated insulin resistance (HOMA-IR) = FINS (mU/L) × FBG (mmol/L)/22.5.

### Real-time PCR quantification of Adipo and AdipoR1

Total skeletal muscle RNA was extracted using TRIzol Reagent (Invitrogen, 15596-026) as described by the manufacturer. cDNA was obtained by reverse transcription of 1 μg of RNA with random primers using PrimeScript RT (Takara, RR055A). qPCR was performed on all samples using an Applied Biosystems 7,300 System and TB Green Premix Ex Taq II (Takara, FY18936). The following primers were used to detect Adipo: (forward: 5′- CCAATGTACCCATTCGCTTTAC-3′; reverse: 5′-GAAGTAGTAGAGTCCCGGAATG-3′), AdipoR1: (forward: GAAAGACAACGACTACCTGCTA; reverse: ATAG CACAAAACCAAGCAGATG), reference gene GAPDH binding protein (forward: 5′-GCACCACCAACTGCTTAG-3′; reverse: 5′- CAGTGATGGCATGGACTG-3′). The ΔΔCT method was used to analyze relative expression levels and statistics were performed using ΔΔCT values.

### Skeletal muscle AdipoR1 protein levels measured by ELISA

Extraction of skeletal muscle tissue was performed as described above. Tissues were then homogenized and the total protein concentration was determined using bicinchoninic acid (BCA), with the concentration not exceeding 0.3 mg. ELISA was performed according to the manufacturer’s instructions (EM0308; Wuhan Fine Biotech Co., Ltd., Wuhan, China).

### Immunofluorescence

For EA treatment, mice were anesthetized and perfused transcardially with 4% paraformaldehyde, and brains were removed and stored in 4% paraformaldehyde overnight. Sagittal brain slices (30 μm) containing the arcuate hypothalamic nucleus (ARC) were prepared on a freezing microtome. After antigen retrieval, the sections were subsequently blocked in phosphate buffered saline (PBS) with 10% normal goat serum and 0.5% Triton X-100 for 1 h at 37°C. POMC neurons and c-Fos were detected using a rabbit anti-POMC antibody (ab254257; abcam; 1:500) and a guinea pig anti-c-Fos antibody (226004; Synaptic System; 1:800) overnight at 4°C. Tissue was then washed in PBS 3 times and incubated with Alexa Fluor 555-conjugated goat anti-rabbit secondary antibody (ab150078; abcam; 1:800) and Alexa Fluor 488-conjugated goat anti-guinea pig (A11073; Invitrogen; 1:500). AdipoR1 was detected using a rabbit anti-AdipoR1 antibody (ab126611; abcam; 1:200) and then incubated with Alexa Fluor 488-conjugated goat anti-rabbit secondary antibody (ab150181; abcam; 1:800). Subsequently, rabbit anti-POMC antibody and Alexa Fluor 555-conjugated goat anti-rabbit secondary antibody were used. Fluorescence imaging was conducted by confocal laser microscopy. Colocalization of POMC neuronal immunoreactivity and c-Fos expression was observed and documented after counting of labeled cells from a total of 16 representative brain slices and every 4 slices were included for each mice.

### Stereotactic surgery

Mice were anesthetized with tribromoethanol (125 mg/kg) injection (i.p.) and fixed on a stereotaxic apparatus. The skull was exposed and holes were drilled in the target area. rAAV-EF1α-DIO-mCherry-WPRE-pA was injected at a rate of 30 nL/min according to the following coordinates: ARC (AP:-1.45; ML: ± 0.22; DV:-5.60). After 5 min, the needle was retracted and the incision was closed with sterile suture. Coordinate positioning and injection were performed by RWD Biotechnology, Shenzhen, China.

### Electrophysiology

Whole cell patch clamp recording and data analysis were performed on POMC neurons prepared from hypothalamus sections. 16-week-old POMC-cre mice were anaesthetized and infused with cold artificial cerebrospinal fluid (ACSF: 25 mM D-glucose, 25 mM NaHCO_3_, 1.25 mM NaH_2_PO_4_^⋅^2H_2_O, 2.5 mM KCl, 0.1 mM MgCl_2_, 127 mM NaCl, 2 mM CaCl_2_^⋅^2H_2_O). Immediately after the brain was removed, it was placed in cold ACSF containing 95% O_2_ and 5% CO_2_. Coronal sections (280 uM) were cut on Leica VT1200S vibrating microtome and incubated for 30 min in oxygen-containing ACSF at 36°C and incubated at room temperature for at least 1 h before recording. Slices were transferred to recording tanks and soaked in oxygen-containing ACSF (34°C) at a flow rate of 2 mL/min. Electrophysiological signals were recorded using a Multi-Clamp 700 B amplifier (Molecular Devices). The resistance of the recording electrode was 5-8 MΩ when filling the solution with sodium k-gluconate. Insulin (2.5 um, HY-P0035, MCE) and Adipo (600 nM, 315-26-100, PeproTech) were added to the recording tank for specific experiments.

### Statistical analyses

Data and statistical analyses were performed using IBM SPSS (version 26.0) and GraphPad Prism (version 8.0.1). All data are presented as mean ± standard error of the mean (SEM). The differences between groups were analyzed by the unpaired *t*-test or one-way analysis of variance (ANOVA) with *post hoc* Bonferroni correction or Tamhane correction, statistical significance was defined as *P* < 0.05 *, *P* < 0.01 ^**^ and *P* < 0.001 ^***^.

## Results and discussion

### EA ameliorate high-fat diet-induced insulin resistance in mice

After 8 weeks of feeding, HFD-fed mice showed significant weight gain (Supplementary Figure 1A), impaired glucose tolerance (Supplementary Figures 1B, C), as well as impaired insulin tolerance (Supplementary Figures 1D, E). Meanwhile, fasting blood glucose (FBG), serum insulin (FINS), and HOMA-IR in the HFD group were considerably increased compared with the CD group (Supplementary Figures 1F–H). These results suggest that HFD-fed induced insulin resistance was successfully established.

**FIGURE 1:**
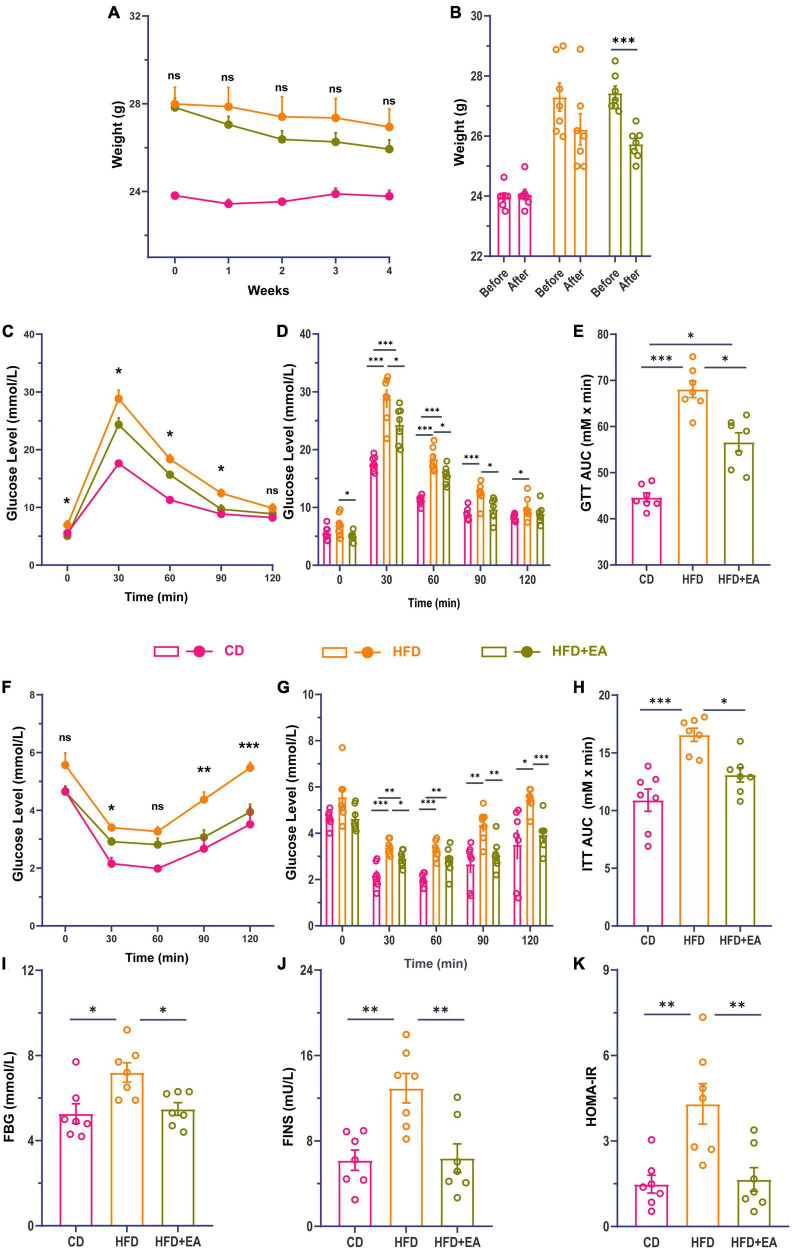
EA ameliorate high-fat diet-induced insulin resistance in mice. **(A)** Comparison of body weight changes during EA. **(B)** Comparison of body weight changes before and after EA. **(C)** Line chart of blood glucose change during glucose tolerance test in mice 4 weeks after EA. **(D)** Histogram of blood glucose change during glucose tolerance test in mice 4 weeks after EA. **(E)** Area under the glucose tolerance curve. **(F)** Line chart of blood glucose change during insulin tolerance test in mice 4 weeks after EA. **(G)** Histogram of blood glucose changes during insulin tolerance test in mice 4 weeks after EA. **(H)** Area under the insulin tolerance curve. **(I)** Fasting blood glucose of mice after EA. **(J)** Fasting serum insulin concentration of mice after EA. **(K)** Insulin resistance index of mice after EA. CD: control group. HFD: high-fat diet group. HFD + EA: model + electroacupuncture group. ^ns^*P* > 0.05; *P < 0.05; ^**^P < 0.01; ^***^P < 0.001 (one-way analysis of variance with *post hoc* Bonferroni correction or Tamhane correction).

After the establishment of our IR model, the HFD group was randomly divided into an HFD group and an HFD + EA group. Following EA intervention for 4 weeks, the HFD + EA group showed a marked trend in weight loss (Figures 1A, B). Compared to the HFD group, the HFD + EA group presented improved glucose and insulin tolerance (Figures 1C–H). Compared with HFD group, the area under the curve (AUC) of ITT in the HFD + EA group decreased significantly, and there was no significant difference between the HFD + EA and CD groups (Figure 1H). FBG, FINS, and HOMA-IR levels were returned to normal levels after EA acupuncture treatment (Figures 1I–K).

### Adipo and AdipoR1 participate in EA-mediated improvement of HFD-IR mice

**FIGURE 2:**
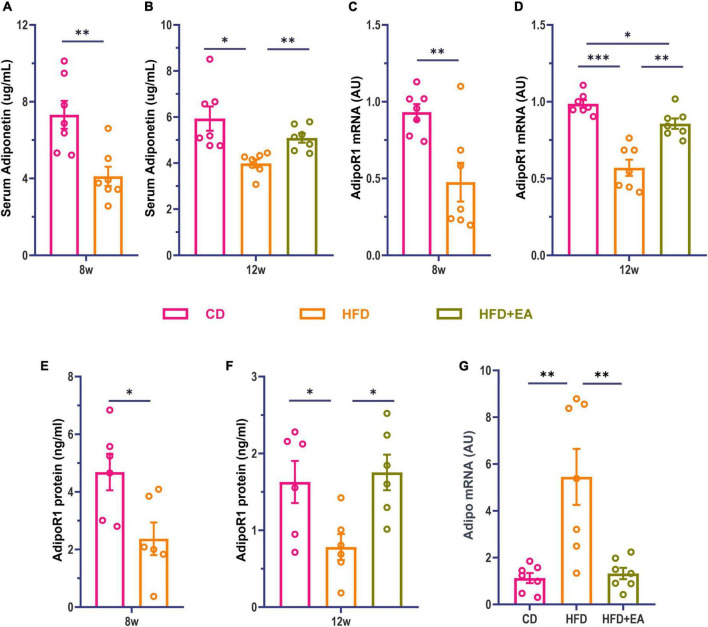
HFD + EA mice exhibited improved levels of Adipo and AdipoR1. **(A)** Serum Adipo concentration of mice after HFD modeling. **(B)** Serum Adipo concentration of mice after EA. **(C)** Expression of AdipoR1 mRNA in skeletal muscle of mice after HFD modeling. **(D)** Expression of AdipoR1 mRNA in skeletal muscle of mice after EA. **(E)** AdipoR1 protein concentration in skeletal muscle of mice after HFD modeling. **(F)** AdipoR1 protein concentration in skeletal muscle of mice after EA. **(G)** Expression of Adipo mRNA in skeletal muscle of mice after EA. CD: control group. HFD: high-fat diet group. HFD + EA: model + electroacupuncture group. ^ns^*P* > 0.05; *P < 0.05; ^**^P < 0.01; ^***^P < 0.001 [unpaired *t*-test in panels **(A,C,E)**; one-way analysis of variance with *post hoc* Bonferroni correction or Tamhane correction in panels **(B,D,F,G)**].

Studies have found that Adipo can increase insulin sensitivity and improve insulin resistance (Fang and Judd, 2018). Adipo receptors include receptor 1 and receptor 2, with receptor 1 mainly distributed in skeletal muscle and receptor 2 in the liver (Borst, 2004; Yadav et al., 2013; James et al., 2021). Although both receptor 1 and receptor 2 may influence glucose metabolism, findings imply that receptor 1 may be more involved in glucose metabolism (Tan et al., 2005). Kadowaki et al. proved that pioglitazone, a commonly used drug for the treatment of type 2 diabetes is likely to play an important role through Adipo (Kubota et al., 2006; Quaresma et al., 2016). Therefore, we investigated whether Adipo was also involved in the improvement of IR by EA. We examined Adipo and AdipoR1 levels in mice, observing that HFD-fed mice were associated with a significant decrease in serum Adipo and skeletal muscle AdipoR1 levels (Figures 2A, C, E). These results suggest that HFD-fed mice develop insulin resistance with decreased Adipo and AdipoR1 levels. After EA treatment, the serum Adipo level of the HFD + EA group was significantly improved with no significant difference compared to CD group (Figure 2B). We then examined the expression levels of AdipoR1 mRNA and protein in skeletal muscle to determine whether AdipoR1 participated in the improvement of IR in the skeletal muscle of mice through EA intervention. Our results showed that AdipoR1 mRNA expression in skeletal muscle of HFD mice had a noteworthy change after EA (Figure 2D). Correspondingly, AdipoR1 protein expression in skeletal muscle was significantly increased in the HFD + EA group and returned to normal levels (Figure 2F). We also detected Adipo mRNA in skeletal muscle. Interestingly, the HFD group showed a significant increase in Adipo mRNA (Figure 2G), which was restored by EA. This result may be related to the compensatory increase of skeletal muscle Adipo in IR conditions. These results suggest that EA treatment can improve insulin resistance in HFD mice, and Adipo and AdipoR1 might contribute to this process.

### EA treatments activate hypothalamic POMC neurons

**FIGURE 3:**
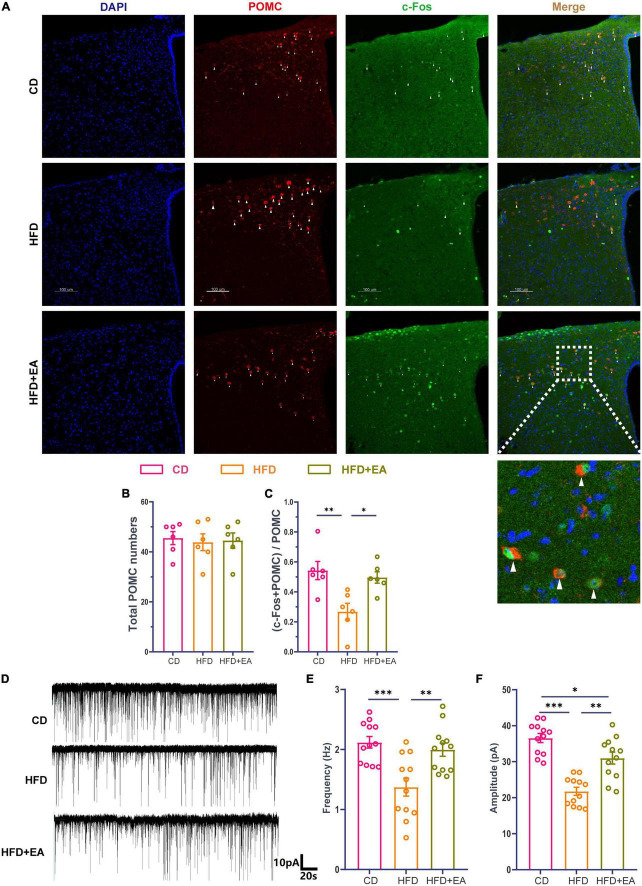
EA treatments activate hypothalamic POMC neurons. **(A)** Co-labeled immunofluorescence representation of POMC neurons and c-Fos. **(B)** Total POMC neurons number. **(C)** Ratio of common standard count of POMC neurons and c-Fos in total POMC neurons. **(D)** Representation of sEPSCs in POMC neurons. **(E)** sEPSCs frequency of POMC neurons. **(F)** sEPSCs amplitude of POMC neurons. “n” in the immunofluorescence graph represents the number of slices, with 3 mice in each group. “n” in the patch clamp graph represents the number of cells (the number of mice in each group ≥ 3). CD: control group. HFD: high-fat diet group. HFD + EA: model + electroacupuncture group. ^ns^*P* > 0.05; *P < 0.05; ^**^P < 0.01; ^***^P < 0.001 (one-way analysis of variance with *post hoc* Bonferroni correction or Tamhane correction). Scale bars: 100 μm.

As observed above, EA treatment of ST36 improved HFD-induced insulin resistance, leading to questions about whether this was related to central regulation. Previous studies have shown that POMC neurons in the arcuate nucleus of the hypothalamus play an important role in regulating metabolism (Schwartz et al., 1992; Sipols et al., 1995; Hill et al., 2010; Zeltser et al., 2012). We wanted to know whether POMC neurons could be activated by EA treatment. Mice were anesthetized 1h after EA treatment and their brains were harvested for immunofluorescence staining. Anti-c-Fos and anti-POMC were jointly labeled (Figure 3A). There was no significant difference in the number of POMC neurons among the three groups (Figure 3B). The activated POMC neurons were significantly enriched in the HFD + EA group compared with the HFD group (Figure 3C) and returned to CD group level. We then examined the electrophysiological properties of POMC neurons using a patch clamp. Cells were clamped at –60mV and sEPSCs were recorded (Figure 3D). In the HFD group, the frequency of sEPSCs decreased meaningfully and the HFD + EA group had rescued this phenomenon and returned to normal level (Figure 3E). Amplitude of sEPSCs showed the same tendency (Figure 3F).

### EA treatments activate hypothalamic POMC neuron responses to Adipo

**FIGURE 4:**
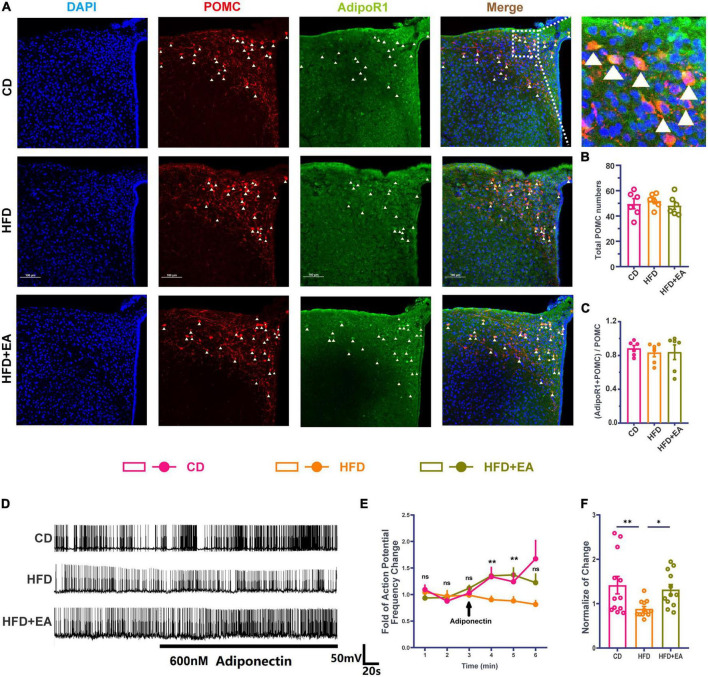
EA treatments activate hypothalamic POMC neurons’ response to Adipo. **(A)** Co-labeled immunofluorescence representation of POMC neurons and AdipoR1 in the arcuate nucleus of the hypothalamus. **(B)** Total POMC numbers. **(C)** Ratio of common standard count of POMC neurons and AdipoR1 in the total POMC number. **(D)** Frequency representation of spontaneous action potential of POMC neurons before and after Adipo perfusion. **(E)** Spontaneous action potential frequency line diagram of POMC neurons before and after Adipo perfusion. **(F)** Normalization of action potential frequency change. “n” in the immunofluorescence graph represents the number of slices, with 3 mice in each group. “n” in the patch clamp graph represents the number of cells (the number of mice in each group ≥ 3). Panel **(E)** shows the statistical results of HFD group and HFD + EA group. CD: control group. HFD: high-fat diet group. HFD + EA: model + electroacupuncture group. ^ns^*P* > 0.05; *P < 0.05; ^**^P < 0.01 (one-way analysis of variance with *post hoc* Bonferroni correction or Tamhane correction). Scale bars: 100 μm.

Although POMC neurons can respond to insulin, mice with an insulin receptor deletion in POMC neurons show normal blood glucose parameters (Hill et al., 2010), suggesting that the effect of insulin on POMC neurons is not the most important factor in improving IR. At the same time, Adipo has been demonstrated to interact directly with POMC neurons under high concentrations of glucose (Suyama et al., 2016), suggesting that the direct effect of Adipo on POMC neurons may be the reason for improving IR. We therefore performed immunofluorescence co-staining in the ARC region of AdipoR1 and POMC neurons (Figure 4A). The result showed no significant difference in the ratio of the co-stained number of AdipoR1 and POMC neurons to the total number of POMC neurons in the CD group, HFD group, and HFD + EA group (Figures 4B, C), suggesting that EA may not improve IR by changing the number of AdipoR1 receptors on POMC neurons.

Pioglitazone (PIO), a diabetes drug, improves the effect of insulin/leptin in the hypothalamus, partly by activating the Adipo/AdipoR1/AMPK axis in the hypothalamus (Quaresma et al., 2016), indicating that Adipo and AdipoR1 may play an important role in improving the effect of hypothalamus IR. Therefore, this research further detected the change of spontaneous action potential frequency of POMC neurons after Adipo perfusion (Figure 4D). In the CD group, Adipo can increase the action potential frequency of POMC neurons. However, POMC neurons cannot respond to Adipo in the HFD group, while EA can restore the response degree of POMC neurons to Adipo (Figure 4E). The normalization of action potential frequency change also indicates that EA can improve the response of POMC neurons to Adipo (Figure 4F). Based on previous studies and the above experiments, we propose a hypothesis that EA ST36 may improve IR through activating POMC neurons responses to Adipo.

## Discussion

In this study, EA at ST36 improved IR induced by high-fat diet while increasing Adipo and AdipoR1 levels. In addition, the activity of POMC neurons and their response to Adipo were weakened in IR and improved after EA. Our results suggest that the enhanced response of POMC neurons to Adipo plays an important role in EA-mediated improvement of IR.

Obesity is closely related to IR, and most obese experimental animals showed impaired insulin response (Al-Massadi et al., 2019; Rodrigues et al., 2019; Zhang F. et al., 2020), while some experimental results demonstrated that FINS concentration in mice fed with a high-fat diet did not change significantly (Hanning et al., 2019). In this study, FBG, FINS, and HOMA-IR increased in model mice, similar to clinical IR. The differences between experimental results and prior research results may be related to the following: (1) dietary nutrient ratio of the mice; (2) Daily caloric intake of the mice; (3) Modeling time; (4) Sampling time. In the streptozotocin- (STZ-) induced insulin-dependent diabetes (IDDM) rat model, Man (Man et al., 2021) found that EA at ST36 can be an effective strategy to reduce plasma glucose and the level of free fatty acids which is one of the major lipids in human body. Ji et al. (2013) concluded that EA ST36 produces an up-regulation of anorexigenic factor POMC production in the NTS/HN, which inhibited food intake and reduced body weight. Meanwhile, Zhang Y. et al. (2020) and Han et al. (2021) also found the similarly result, which suggest that single point stimulation at ST36 can reduce body weight. In this experiment, it was observed that the body weight of the HFD + EA group only a decreased compared with the HFD group during EA, suggesting that a single EA treatment could not restore the body weight of model mice to a normal level. In intragroup comparison, it was observed that the body weight of the HFD + EA group decreased significantly compared to before EA, and there was no significant difference in body weight between the HFD group and the CD group. This may be related to the small size of the mice themselves and the insignificant weight change. Although weight gain is inextricably linked to IR, weight is just one of the manifestations of IR, and IR may also can occur without high body mass index (BMI) (Brown and Walker, 2016). In clinical practice, weight control is often combined with exercise and drugs to reduce weight (Bray et al., 2016; Lundgren et al., 2021). Therefore, we propose that acupuncture at a single point may not be the ideal method to improve body weight of IR individuals. In this study, EA could improve IPGTT, IPITT, FBG, FINS levels, and HOMA-IR in IR mice, which was consistent with the results of others (Yi et al., 2007; Chen et al., 2017; Huang et al., 2019).

The improvement of IR by acupuncture may be related to the improvement of glucose transport capacity in the hypothalamus (Hong et al., 2017). POMC neurons located in the ARC region of the hypothalamus are key neurons in regulating metabolism and energy. Some studies suggest that POMC neurons show different responses to different concentrations of glucose (Suyama et al., 2016; Fioramonti et al., 2017), indicating that insulin may also have similar effects on POMC neurons. At the same time, POMC neurons are innervated by other neurons (Newton et al., 2013; Trotta et al., 2020). In this research, EA was observed to improve the expression of c-Fos in POMC neurons, which may be related to the speed of releasing the transmitter from the presynaptic membrane of POMC neurons and the changes in the number of receptors in the postsynaptic membrane.

Skeletal muscle is the main target of Adipo and a major location of AdipoR1. Although skeletal muscle is considered to be one of the targets of pharmacological treatment for IR or diabetes (Dong et al., 2021; Wei et al., 2021; Yu et al., 2022), some studies have shown that acupuncture ST36 may stimulate skeletal muscle and improve the recovery of behavioral activity in rats subjected to cerebral ischemia or reperfusion injury (Yang et al., 2022). Sun demonstrated that acupuncture at ST36 increased motor cortical excitation (Sun et al., 2019). The deep layer of ST36 is skeletal muscle, and ST36 is the only acupuncture point selected in this experiment. Therefore, skeletal muscle probably will be one of the targets of acupuncture treatment for IR or diabetes. Based on previous studies (da Silva Rosa et al., 2021), we know that there is a degradation of Adipo during insulin resistance, which can also lead to a decrease in Adipo levels and thus an inability to maintain normal insulin sensitivity. Meanwhile, our research indicates that the expression levels of AdipoR1 mRNA and protein in skeletal muscle of the HFD group were significantly decreased, suggesting that the ability of skeletal muscle to process Adipo in IR was impaired, which may be one of the reasons for decreased insulin sensitivity in model mice. EA at ST36 heightened the expression of AdipoR1 mRNA and protein in skeletal muscle of HFD group mice and rescued Adipo levels in both serum and skeletal muscle.

Pioglitazone (PIO), a diabetes drug, improves the effect of insulin/leptin in the hypothalamus, partly by activating the Adipo/AdipoR1/AMPK axis in the hypothalamus (Quaresma et al., 2016), suggesting that Adipo and AdipoR1 may play an important role in improving the effect of IR in hypothalamus. Therefore, this experiment further demonstrated the change of spontaneous action potential frequency of POMC neurons after Adipo perfusion. Under physiological conditions, Adipo can increase the discharge frequency of POMC neurons, and this function is reduced in the IR pathological state, while EA can restore the response degree of POMC neurons to Adipo. According to previous research, the peripheral injections (Kandasamy et al., 2012) or intraportal injection (Shklyaev et al., 2003) of lipocalin encoding vectors can drive expression of lipocalin cDNA, which would suggest that muscle-derived lipocalin would be secreted into the bloodstream thereby increasing serum lipocalin levels, while acting to reduce body weight and food intake (Tang et al., 2021). At the same time, there are some viewpoints that peripheral adiponectin can be transferred to the brain through the blood-brain barrier (Koch et al., 2014) and the expression of adiponectin in hypothalamic mHypoPOMC and SH-SY5Y neurons and their regulation at the neuronal level (Abgrall et al., 2022). Based on previous studies and the above experiments, we propose a hypothesis that EA at ST36 may regulate Adipo through POMC neurons, thereby improving IR. However, there are still other neurons in this location that may impact IR in addition to POMC neurons. Studies have shown that Adipo can independently enhance the inhibitory postsynaptic current of NPY neurons at the physiological level (Suyama et al., 2017), and glucose levels may influence the excitatory or inhibitory effect of Adipo on the activity and feeding of arcuate POMC neurons. In subsequent experiments, we will specifically regulate AdipoR1 on POMC neurons to observe the role of POMC neurons in regulating Adipo in IR.

## Conclusion

This study investigated the effect of EA at ST36 on IR in peripheral and central functions. Our study illustrates the possibility that EA regulates metabolism and energy through the response of POMC neurons to Adipo. Future research should explore the underlying mechanisms of intrinsic excitatory changes and how central signals are transmitted to the periphery. In addition, further research is needed on central neurons that regulate metabolism and energy.

## Data availability statement

The original contributions presented in this study are included in the article/Supplementary material, further inquiries can be directed to the corresponding author.

## Ethics statement

This animal study was reviewed and approved by the Ethics Committee of Guangzhou University of Chinese Medicine (No. 20210604003).

## Author contributions

WX: study design, performance of the experiments, data analysis, statistical analysis, and writing and revision of the manuscript. JL: performance of the animal feeding work, assistance with the experiments, and writing and revision of the manuscript. CJ: study design and assistance with the molecular experiments. DF: assistance with the animal feeding work and molecular experiments. LY and NX: study design and revision of the manuscript. WY: study design and writing and revision of the manuscript. All authors contributed to the article and approved the submitted version.
