# Supplementary figures and images for: Activation of POMC neurons to adiponectin participating in EA-mediated improvement of high-fat diet IR mice
Source: Front Neurosci. 2023 Mar 21;17:1145079. doi: 10.3389/fnins.2023.1145079 (PMC10077892; doi:10.3389/fnins.2023.1145079)

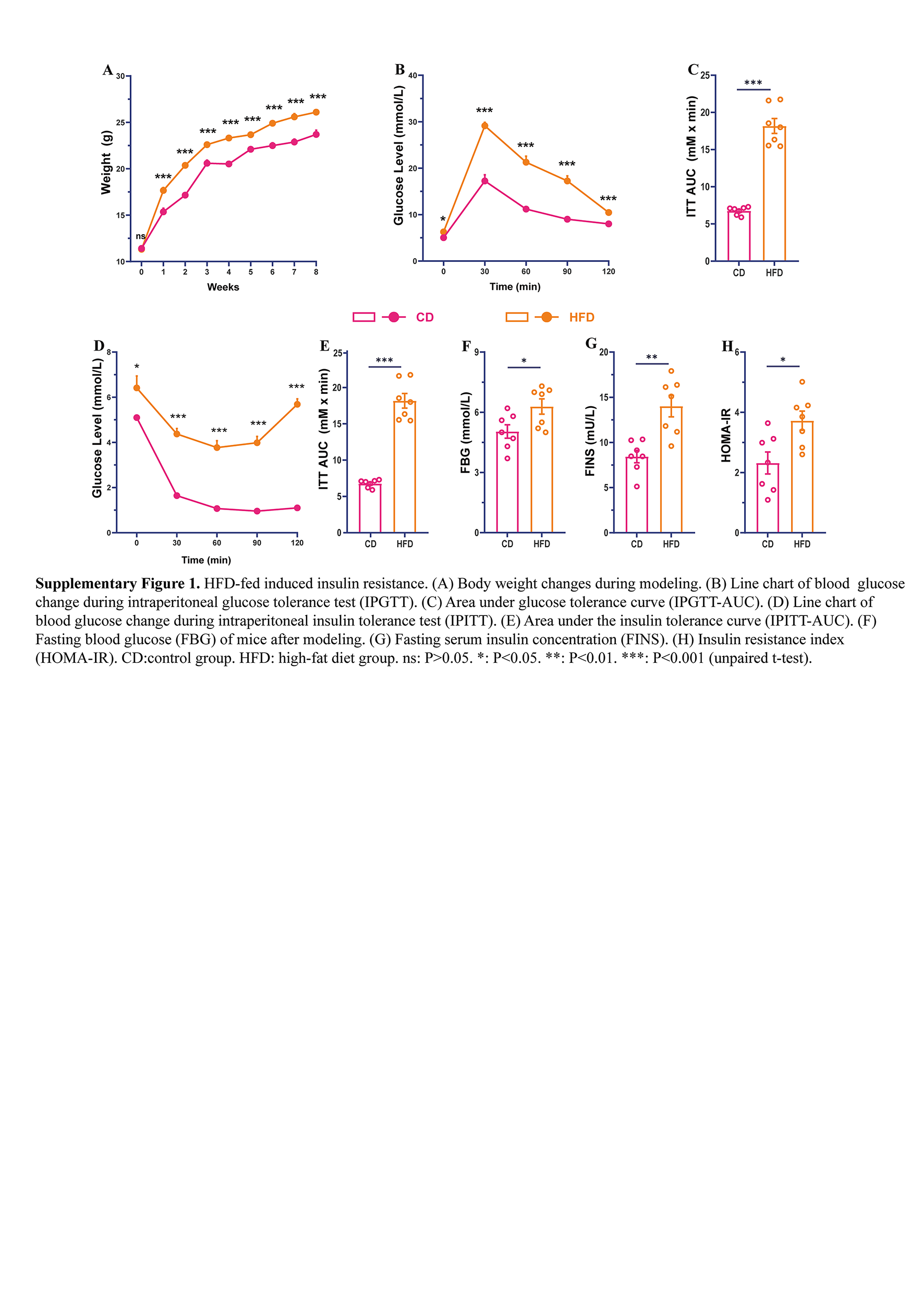

Supplement: Supplementary file 1 [file Image_1.tif]

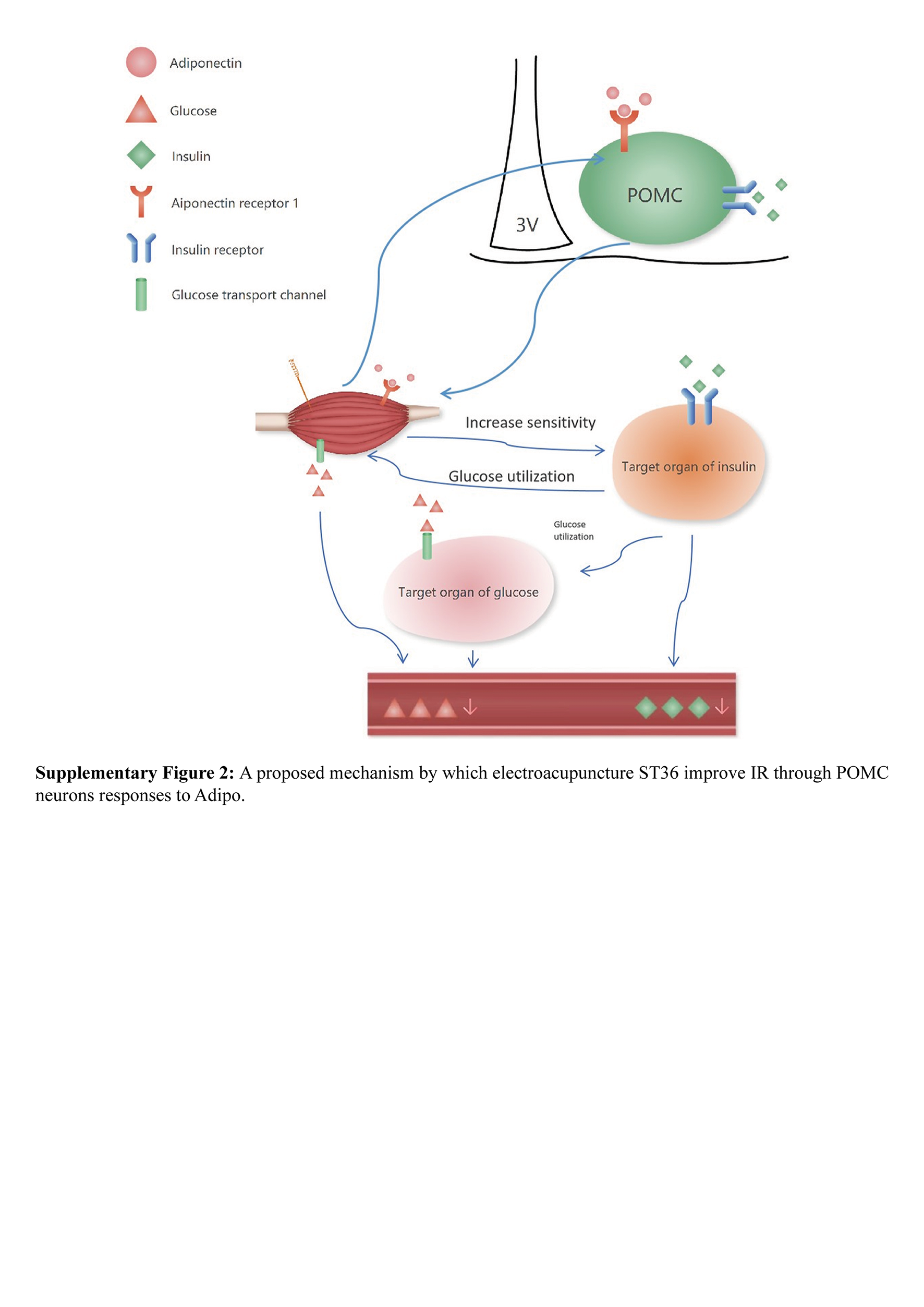

Supplement: Supplementary file 2 [file Image_2.tif]
